# A Contraction Stress Model of Hypertrophic Cardiomyopathy due to Sarcomere Mutations

**DOI:** 10.1016/j.stemcr.2018.11.015

**Published:** 2018-12-13

**Authors:** Rachel Cohn, Ketan Thakar, Andre Lowe, Feria A. Ladha, Anthony M. Pettinato, Robert Romano, Emily Meredith, Yu-Sheng Chen, Katherine Atamanuk, Bryan D. Huey, J. Travis Hinson

**Affiliations:** 1 The Jackson Laboratory for Genomic Medicine, 10 Discovery Drive, Farmington, CT 06032, USA; 2 University of Connecticut School of Medicine, 263 Farmington Avenue, Farmington, CT 06032, USA; 3 Department of Biomedical Engineering, University of Connecticut, Storrs, CT 06269, USA; 4 Department of Materials Science and Engineering, University of Connecticut, Storrs, CT 06269, USA

**Keywords:** induced pluripotent stem cells, cardiomyopathy, heart failure, tissue engineering, sarcomere function, hypertrophyp53 signaling

## Abstract

Thick-filament sarcomere mutations are a common cause of hypertrophic cardiomyopathy (HCM), a disorder of heart muscle thickening associated with sudden cardiac death and heart failure, with unclear mechanisms. We engineered four isogenic induced pluripotent stem cell (iPSC) models of β-myosin heavy chain and myosin-binding protein C3 mutations, and studied iPSC-derived cardiomyocytes in cardiac microtissue assays that resemble cardiac architecture and biomechanics. All HCM mutations resulted in hypercontractility with prolonged relaxation kinetics in proportion to mutation pathogenicity, but not changes in calcium handling. RNA sequencing and expression studies of HCM models identified p53 activation, oxidative stress, and cytotoxicity induced by metabolic stress that can be reversed by p53 genetic ablation. Our findings implicate hypercontractility as a direct consequence of thick-filament mutations, irrespective of mutation localization, and the p53 pathway as a molecular marker of contraction stress and candidate therapeutic target for HCM patients.

## Highlights

• We engineered four iPSC models of HCM with *MYH7* and *MYBPC3* mutations with CRISPR
• HCM mutations result in hypercontractility in 3D cardiac microtissues
• Hypercontractility was independent of changes in calcium handling
• HCM cardiomyocytes exhibit oxidative stress and p53-dependent cytotoxicity with stress

## Teaser

Cohn et al. show that thick-filament sarcomere mutations that cause hypertrophic cardiomyopathy result in hypercontractility in human cardiac microtissues engineered from isogenic iPSCs. These findings illustrate that hypercontractility is independent from changes in calcium handling and mutation location, but results in oxidative stress, p53 activation, and increased p53-dependent cell death with metabolic stress.

## Introduction

**Figure 1:**
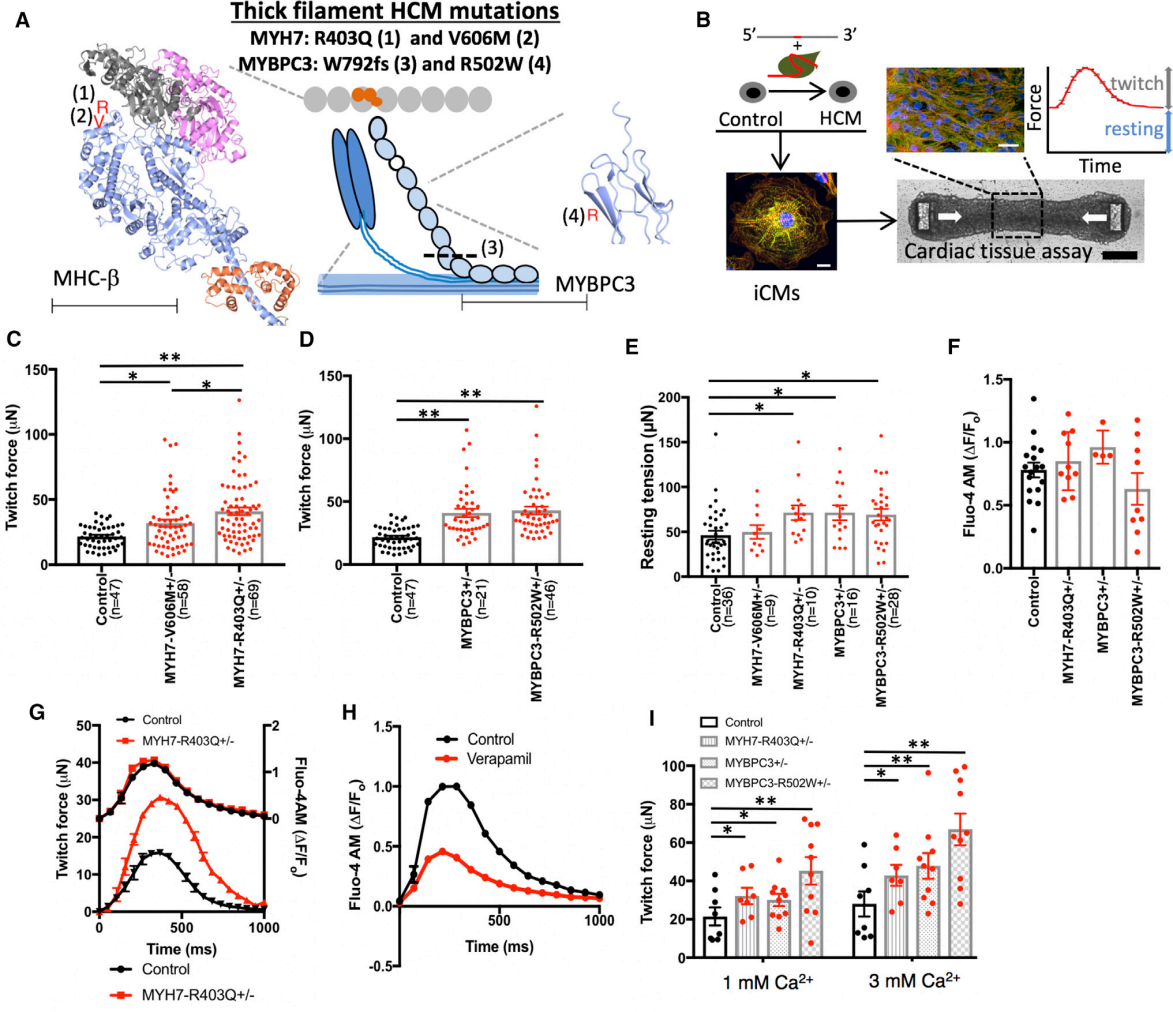
Human iPSC-Derived CMT Models with Thick-Filament HCM Mutations Result in Hypercontractility (A) A representation of the sarcomere is shown that includes thick-filament components myosin heavy chain β (MHC-β) (blue globular heads connected to thin rods) and myosin-binding protein C (MYBPC3) (chain of light blue ovals); and thin-filament components actin (gray ovals) and the troponin complex (orange ovals). Location of mutations are decorated on the crystal structures of MHC-β-S1 (blue ribbon, left) and a domain of MYBPC3 (blue ribbon, right) (Fujii and Namba, 2017). Note: MHC-β-S1 is shown interacting with two actin molecules (gray and pink ribbons) and a regulatory light chain (orange ribbon). For MYH7, R403Q is identified by a red R (1), and V606M is denoted by a red V (2). For MYBPC3, the location of the truncation W792fs is denoted by a dashed line (3), and R502W is denoted by a red R (4). Scale bars, 62.5 Å (MHC-β) and 31 Å (MYBPC3). (B) Experimental outline of isogenic HCM model generation using the guide RNA (gRNA)/Cas9 complex and single-stranded oligodeoxynucleotide to introduce HCM mutations into a control iPSC line. iPSCs are then differentiated to produce iCMs that are combined with fibroblasts and an extracellular matrix slurry for CMT production. Scale bar, 10 μm. White arrows depict direction of contraction. Scale bars, 25 μm (top panel) and 200 μm (bottom panel). Both tissue twitch force and resting tension are quantified as well as CMT sarcomere structure by immunofluorescence. (C) Maximum twitch force from CMTs generated from control, MYH7-V606M^+/–^ and MYH7-R403Q^+/–^ iCMs. (D) Maximum twitch force from CMTs composed of control, MYBPC3^+/−^, and MYBPC3-R502W^+/–^ iCMs. (E) Resting tension produced by HCM CMTs compared with controls. (F and G) Quantification of calcium transients (ΔF/F_o_) measured in HCM and control CMTs stained with Fluo-4 while pacing at 1 Hz (F). See representative tracing in (G). (H) Representative calcium transient tracing of control iCMs treated with verapamil or carrier control. (I) Dependence of maximum twitch force generated by HCM and control CMTs from extracellular calcium concentration. Significance assessed by ANOVA (C–F and I) (^∗^all p < 0.05 and ^∗∗^all p < 0.001); data are means ± SEM (error bars) (C–I). Each data point represents a single CMT (C–F and I) generated by at least three biological replicates by iPSC differentiation batch.

Hypertrophic cardiomyopathy (HCM) is a human disorder that affects 1 in 500 individuals with uncertain mechanisms (Maron et al., 1995). Patients with HCM are diagnosed by the presence of unexplained left ventricular hypertrophy (LVH) with preserved systolic contractile function (Ho et al., 2002). In young athletes, HCM manifests as a common cause of sudden cardiac death; while, in adults, HCM is associated with heart failure that may progress to require cardiac transplantation (Gersh et al., 2011b). Over the last few decades, the genetic basis of HCM has been demonstrated by inheritance of autosomal dominant mutations in components of the force-producing sarcomere (Maron et al., 2012). About two-thirds of HCM patients harbor heterozygous mutations in one of two sarcomere genes: myosin heavy chain β (MHC-β is encoded by *MYH7*) or myosin-binding protein C (cMyBP-C is encoded by *MYBPC3*) (Maron et al., 2012). Along with titin, MHC-β and cMyBP-C are located in the thick filament where ATP hydrolysis by MHC-β is coupled to force generation through interactions with the actin-rich thin filament (Figure 1A). A prevailing model suggests that HCM mutations alter cardiac force generation through dysregulation of calcium handling (Ashrafian et al., 2011, Lan et al., 2013). Whether *MYBPC3* and *MYH7* mutations result in HCM by shared or heterogeneous mechanisms remains undetermined.

Recent functional studies of thick-filament HCM mutations in reconstituted sarcomere and cardiomyocyte assays have supported both gain- and loss-of-force production models of HCM pathogenesis, thus suggesting that changes in force production may not be a shared consequence of HCM mutations. For example, MYH7-R453C (arginine 453 substituted with cysteine) increased while MYH7-R403Q (arginine 403 substituted with glutamine) decreased force production in reconstituted actomyosin motility assays (Nag et al., 2015, Sommese et al., 2013). Equally puzzling, contractile studies of single cardiomyocytes from MYH6-R403Q^+/–^ mouse models, which recapitulate LVH and fibrosis *in vivo* (Geisterfer-Lowrance et al., 1990), have produced similarly conflicting results for the identical mouse model and strain (Chuan et al., 2012, Kim et al., 1999). Human patient-specific induced pluripotent stem cell (iPSC) HCM models of MYH7-R663H (arginine 663 substituted with histidine) have recapitulated some features of HCM including cellular enlargement and altered calcium handling (Lan et al., 2013), but mechanical phenotypes of HCM iPSC models have not been comprehensively studied.

The apparent difficulty in establishing the pathogenesis of HCM has been attributed in part to: (1) multiprotein assembly limitations that hinder sarcomere functional analysis, (2) mouse models that express distinct sarcomere components compared with humans (e.g., *MYH6* instead of *MYH7*), (3) the lack of isogenic iPSC-derived cardiomyocyte cell lines to control for genetic and epigenetic variation, and (4) the absence of biomimetic 3D human cardiac tissue functional assays. Here, we set out to address these limitations by combining genetic engineering tools to generate a series of scarless *MYH7* and *MYBPC3* HCM mutations in human isogenic iPSCs that are differentiated to cardiomyocytes (iCMs) that express human sarcomere contractile components. We generated 3D cardiac microtissues (CMTs) (Figure 1B) to identify mechanical consequences of HCM mutations in combination with molecular assays to interrogate insights into HCM pathogenesis.

## Results

### Generation of HCM iPSC and CMT Models Using CRISPR/Cas9

We began by identifying two HCM mutations in *MYH7*, R403Q and V606M (valine 606 substituted with methionine), which cause autosomal dominant HCM in both humans (Geisterfer-Lowrance et al., 1990, Marian et al., 1995) and mice (Blankenburg et al., 2014, Geisterfer-Lowrance et al., 1996). Both mutations are located in subfragment 1 (S1) of MHC-β near the actin-interacting domain (Figure 1A), but R403Q leads to a more severe cardiomyopathy compared with V606M (Blankenburg et al., 2014). Because MHC-β physically interacts with MYBPC3 (Maron et al., 2012), we hypothesized that these mutations could lead to HCM by a shared mechanism.

We next selected a common pathogenic truncation mutation in *MYBPC3* (clinvar.com), a guanine insertion that leads to tryptophan 792 substituted with valine followed by a frameshift (Trp792ValfsX41; MYBPC3^+/−^), and a common pathogenic missense mutation, arginine 502 substituted with tryptophan (R502W) that is found in up to 2.4% of HCM patients (Figure 1A) (Saltzman et al., 2010). We generated scarless, isogenic iPSC models of these four mutations with CRISPR technology using delivery of a single optimized guide RNA, Cas9 nuclease, and a single-stranded oligodeoxynucleotide repair template (Figure 1B; Table S1). We chose an isogenic approach to focus our investigation on the direct functional consequences of the four thick-filament mutations, while controlling for background epigenetic and genetic variation. To generate the four iPSC models, we screened 1,111 clones by Sanger sequencing (Table S1). After screening for off-target genome-editing loci by sequencing (crispr.mit.edu) and karyotype abnormalities by virtual karyotyping using arrays (Table S2), we directly differentiated iPSCs (Lian et al., 2013) followed by metabolic enrichment (Tohyama et al., 2013) to generate purified iCMs. Finally, we combined iCMs with tissue-forming fibroblasts and an extracellular matrix slurry to generate a 3D CMT assay that recapitulates native cardiac architecture and mechanics, which has been adapted from prior assays applied to study contractility phenotypes of dilated and PRKAG2 cardiomyopathy iPSC models (Boudou et al., 2012, Hinson et al., 2015, Hinson et al., 2016).

### Thick-Filament HCM Mutations Result in Hypercontractility

To study the mechanical consequences of *MYH7* and *MYBPC3* mutations in a biomimetic context, we first optimized our CMT assay (Hinson et al., 2015, Hinson et al., 2016) to increase sarcomere gene expression of thick-filament transcripts. In particular, *MYH7*:*MYH6* is upregulated in the developing human heart (Wessels et al., 1991) and iCMs express fetal- to neonatal-stage transcript levels. By increasing the size of the microfabricated tissue gauges, including a proportional increase in cantilever dimensions and spring constant (Figure S1A), we improved CMT durability from 4 to 10 days (Figure S1B), which was associated with a 42% increase in *MYH7*:*MYH6* expression (Figure S1C, left panel) and 630% increase in cardiac troponin I (*TNNI3*) expression (Figure S1C, right panel). We utilized this improved CMT assay to study HCM mutations. For all CMT studies, we measured contractility parameters on day 7 after tissue compaction has completed and force production has plateaued (Figure S1D).

CMTs generated from iCMs with MYH7-R403Q^+/–^ (Figure S1E) generated a 40.9-μN twitch force compared with 21.7 μN in isogenic controls (Figure 1C). We then tested whether the increased twitch force generated by R403Q^+/–^ was secondary to changes in sarcomere isoform expression or clonal variation. *TNNI3* expression (Yang et al., 2014), a transcript marker that is related to cardiomyocyte maturation, was unchanged (Figures S2A and S2B) (Yang et al., 2014). Increased contraction force was also similar between two independent R403Q^+/–^ clones (Figure S2C). Moreover, CMT cross-sectional area did not differ between HCM mutations and isogenic controls (Figure S2D), which confirmed that HCM tissues were not hypercontractile secondary to increased CMT thickness. We next tested whether CMT assays could predict pathogenicity of *MYH7* mutations by testing CMTs generated from iCMs with the less-pathogenic *MYH7* variant V606M^+/–^, which is also located in the actin-binding domain of S1 (Figure S1F). V606M^+/–^ CMTs generated a 31.9-μN twitch force compared with 21.7 μN in isogenic controls (Figure 1C), which was less than R403Q^+/–^. We concluded that *MYH7* variant pathogenicity positively correlates with maximum contraction force in CMT assays for the two mutations tested. We also generated two *MYBPC3* mutant CMT models to compare with *MYH7* models. MYBPC3^+/−^ and MYBPC3-R502W^+/–^ CMTs generated a 41.0- and 42.9-μN twitch force compared with 21.7 μN in isogenic controls, respectively (Figure 1D and Videos S1 and S2). Because sarcomere function also contributes to twitch-independent contraction force, or resting tension, we measured this parameter in CMTs. In parallel to twitch force changes, resting tension was increased in all HCM models except for MYH7-V606M^+/–^ (Figure 1E). Because V606M^+/–^ results in a mild phenotype, we did not further characterize this variant.

Video S1. Isogenic Control CMT Paced at 1 Hz

Video S2. HCM (MYBPC3-R502W^+/–^) CMT Paced at 1 Hz

To characterize the molecular basis of HCM hypercontractility, we measured both calcium transients and levels in CMTs by optical imaging and calcium-dependent fluorescent dyes. HCM-associated hypercontractility was not related to changes in calcium delivery to the myofilament as calcium transients were unaffected in all HCM mutations tested (Figures 1F and 1G). This is distinct from CMT treatment with verapamil, a voltage-gated calcium channel blocker, which resulted in diminished CMT force production in parallel to reduced calcium transients as expected (Figure 1H). Because the increase in resting tension observed in HCM CMTs could be secondary to myofilament activation due to increased resting calcium levels, we stained iCMs with Indo-1 and measured both resting and caffeine-induced activation. Indo-1 signal was unchanged in HCM CMTs compared with controls at baseline and after caffeine treatment (Figures S2E and S2F). Finally, we tested CMT force production at low and high calcium concentrations (1 and 3 mM) to address the dependence of extracellular calcium on HCM hypercontractility. We found that at both low (1 mM) and high (3 mM) calcium levels, HCM CMTs compared with controls resulted in hypercontractility (Figure 1I). These data support a model whereby thick-filament HCM mutations result in hypercontractility that is independent of variant localization, myofilament calcium delivery, and extracellular calcium levels.

**Figure 2:**
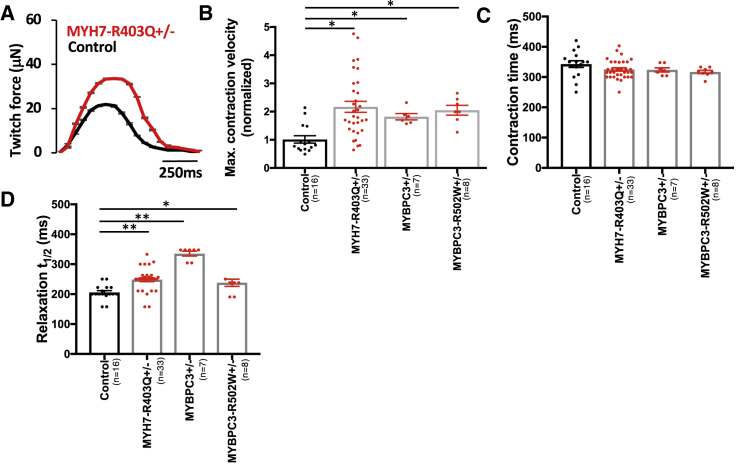
Contraction and Relaxation Kinetics Are Altered by HCM Mutations (A) Representative twitch force tracings from MYH7-R403Q^+/–^ CMT models compared with isogenic controls. (B–D) Normalized maximum contraction velocity (B), contraction time (C), and relaxation half-time (t_1/2_) (D) for MYBPC3^+/−^, MYBPC3-R502W^+/–^, and MYH7-R403Q^+/–^ compared with controls. Significance was assessed by ANOVA (B–D) (^∗^all p < 0.05 and ^∗∗^all p < 0.001); data are means ± SEM (error bars) (A–D). Each data point represents a single CMT (B–D) generated by at least three biological replicates by iPSC differentiation batch.

We also measured HCM-associated contraction and relaxation kinetics by quantifying maximum contraction velocity, contraction time, and relaxation half-time across all HCM mutations. Compared with controls, HCM CMTs exhibited a 79%–121% increased maximum contraction velocity (Figures 2A and 2B), but without changes in contraction time (Figure 2C). Relaxation half-time (t_1/2_) was prolonged (Figure 2D), which further supports that impaired relaxation is a consequence of HCM thick-filament mutations, which has also been documented in HCM patients (Ho et al., 2017). These kinetic changes are distinct from kinetic changes induced by omecamtiv mecarbil (Malik et al., 2011), a direct myosin activator that prolongs contraction time. We conclude that HCM mutations induce a state of hypercontractility that is distinct from how omecamtiv mecarbil activates MHC-β through stabilization of the lever arm (Planelles-Herrero et al., 2017).

### Reduction of Hypercontractility by Calcium Channel Blockers and Direct Myosin Inhibitors

**Figure 3:**
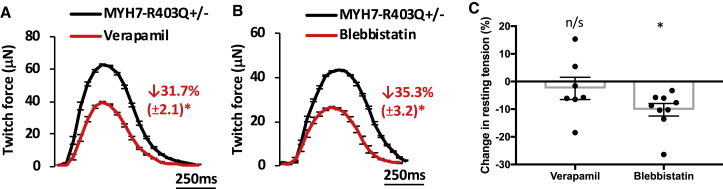
Reduction of Hypercontractility in MYH7-R403Q^+/–^ Tissues by Small Molecules Representative tracings and percentage change in twitch force for MYH7-R403Q^+/–^ CMTs treated with carrier control (black tracing) compared with (A) verapamil (0.5 μM; red tracing) or (B) blebbistatin (10 μM; red tracing). (C) Comparison of the effects of verapamil and blebbistatin on resting tension in MYH7-R403Q^+/–^ CMTs. Significance was assessed by Student's t test (A–C) (^∗^all p < 0.05); data are means ± SEM (error bars) (A–C). Each data point represents a single CMT (C) generated by at least three biological replicates by iPS differentiation batch.

We generated CMTs from the severe HCM mutation MYH7-R403Q^+/–^, and assessed changes in twitch force and resting tension after treatment with two candidate small molecules. We started by evaluating one of the most commonly prescribed therapies for HCM patients, the voltage-dependent L-type calcium channel blocker verapamil (Gersh et al., 2011a, Endoh and Blinks, 1988). In accord with changes in calcium transients (Figure 1H), the addition of verapamil reduced twitch tension by 31.7% in R403Q^+/–^ CMTs (Figure 3A). We also tested the direct myosin inhibitor blebbistatin to assess whether myosin inhibitors may similarly reduce HCM-associated hypercontractility in R403Q^+/–^ CMTs. Since blebbistatin binds to MHC-β distinct from residues near R403Q, and does not interfere with actin-binding or actomyosin disassociation (Kovacs et al., 2004), we hypothesized that this molecule may also reduce twitch force. Similar to verapamil, blebbistatin reduced twitch force by 35.3% (Figure 3B). Because blebbistatin inhibits myosin by a calcium-independent mechanism and resting calcium levels were not altered by HCM mutations, we hypothesized that only blebbistatin could normalize both twitch and resting tension induced by HCM mutations. Indeed, blebbistatin but not verapamil reduced resting tension by 10.2% (Figure 3C). These data obtained from R403Q^+/–^ CMTs suggest that therapeutic agents that reduce both twitch and resting forces may be more efficacious for HCM patients, which may explain why verapamil has limited clinical efficacy in HCM patients (Sen-Chowdhry et al., 2016).

### MYH7-R403Q^+/–^ CMTs Exhibit Myofibrillar Disarray and iCM Hypertrophy

**Figure 4:**
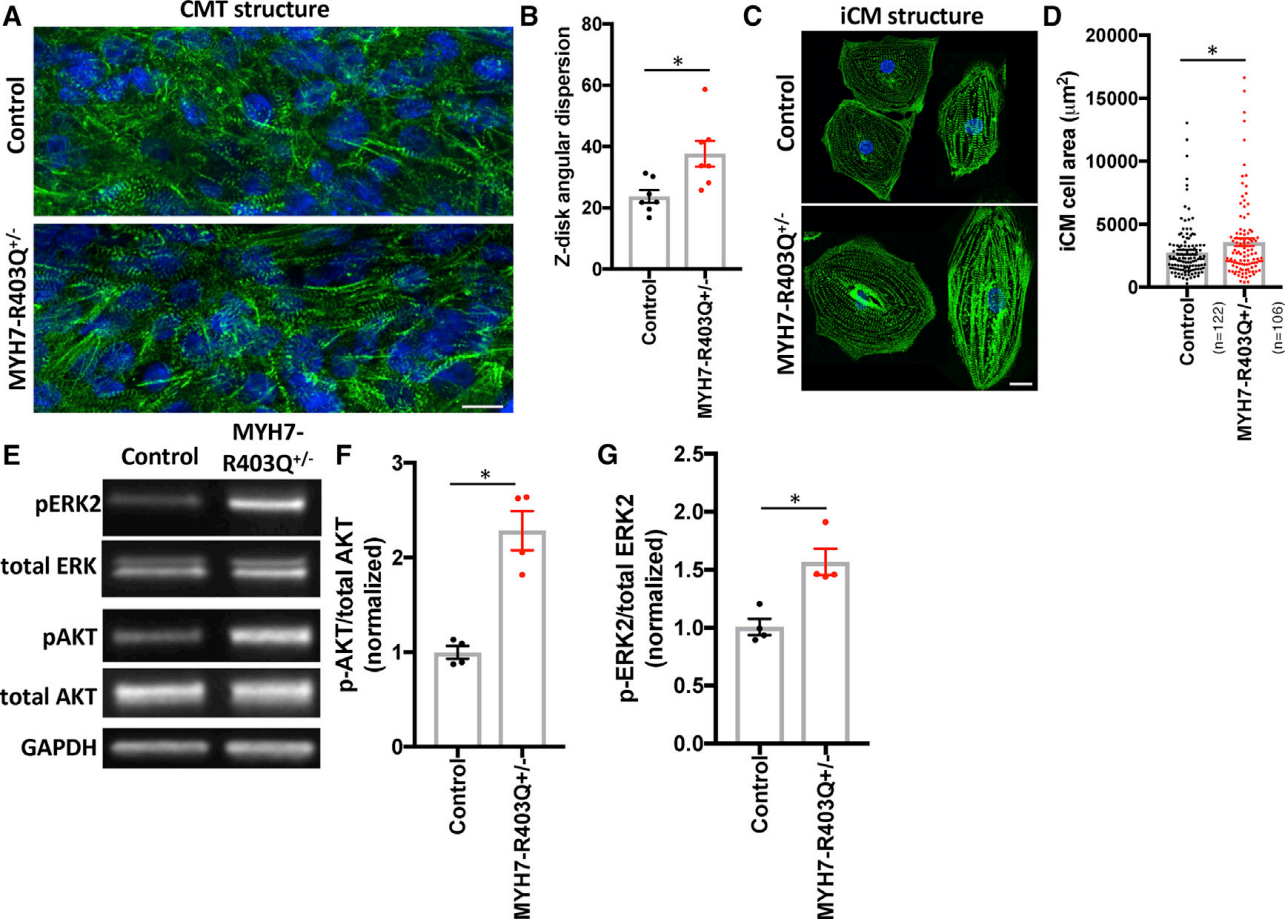
CMT and iCM Structural and Molecular Signaling Changes in MYH7-R403Q^+/–^ Models (A) Representative confocal image of CMTs generated from MYH7-R403Q^+/–^ and control iCMs and decorated with antibodies to cardiac alpha actinin (green) and co-stained with DAPI (blue). Scale bar, 10 μm. (B) Quantification of sarcomeric Z disk angular dispersion obtained from analysis of confocal regions of interest from CMTs decorated with antibodies to alpha actinin. (C) Representative confocal images of single MYH7-R403Q^+/–^ and control iCMs decorated with antibodies to cardiac alpha actinin (green) and co-stained with DAPI (blue). Scale bar, 10 μm. (D) Quantification of MYH7-R403Q^+/–^ and control iCM cell area from confocal images stained with alpha actinin. (E–G) Representative immunoblots from MYH7-R403Q^+/–^ and control iCM lysates probed with antibodies to phospho- and total ERK (note: ERK2 is highly phosphorylated in iCM lysates) and phospho- and total AKT as well as GAPDH (loading control) (E). Normalized quantification of (F) phospho-AKT to total AKT and (G) phospho-ERK2 to total ERK2. Significance was assessed by Student's t test (B, D, F, and G) (^∗^all p < 0.05); data are means ± SEM (error bars) (B, D, F, and G). Each data point represents a single CMT (B), iCM (D), or sample generated from a batch of iCMs (F and G) generated by at least three biological replicates by iPS differentiation batch.

To determine whether HCM mutations affect sarcomere structure in CMTs, we fixed CMTs and stained sarcomeres using antibodies to Z-disc protein alpha actinin with DAPI co-stain (Figure 4A). We again focused on R403Q^+/–^ because this mutation is highly pathogenic in humans and in our mechanical assays. Z disk analysis of R403Q^+/–^ tissues demonstrated increased Z disk angular dispersion (Figures 4B and S3A), which is a measure of Z disk disarray. This result is consistent with myofibrillar disarray observed in HCM patients (Bulkley et al., 1977). Sarcomere length and cell number were not different (Figures S3B and S3C) in R403Q^+/–^ CMTs compared with isogenic controls. To assess iCM size, we fixed and immunostained R403Q^+/–^ iCMs (Figure 4C) because the analysis of individual iCMs is not feasible within our CMT assays. Single R403Q^+/–^ iCMs demonstrated increased iCM cell area (Figure 4D). We next measured levels of candidate hypertrophy signaling pathways including mitogen-activated protein kinase pathways, AKT and CAMKII, as these have been implicated in cardiomyocyte hypertrophy *in vivo* and in iCMs (Hinson et al., 2015, Zhang et al., 2003). In proportion to increased iCM cell area, lysates from R403Q^+/–^ iCMs had elevated levels of phosphorylated ERK2 and AKT (Figures 4E–4G), but not p38, JNK, or CAMKII (Figure S3D). In summary, R403Q^+/–^ mutations result in sarcomere disorganization in CMT assays, and iCM hypertrophy in parallel with increased ERK2 and AKT signaling.

### RNA Sequencing Identifies Activation of p53 Signaling in HCM Models

**Figure 5:**
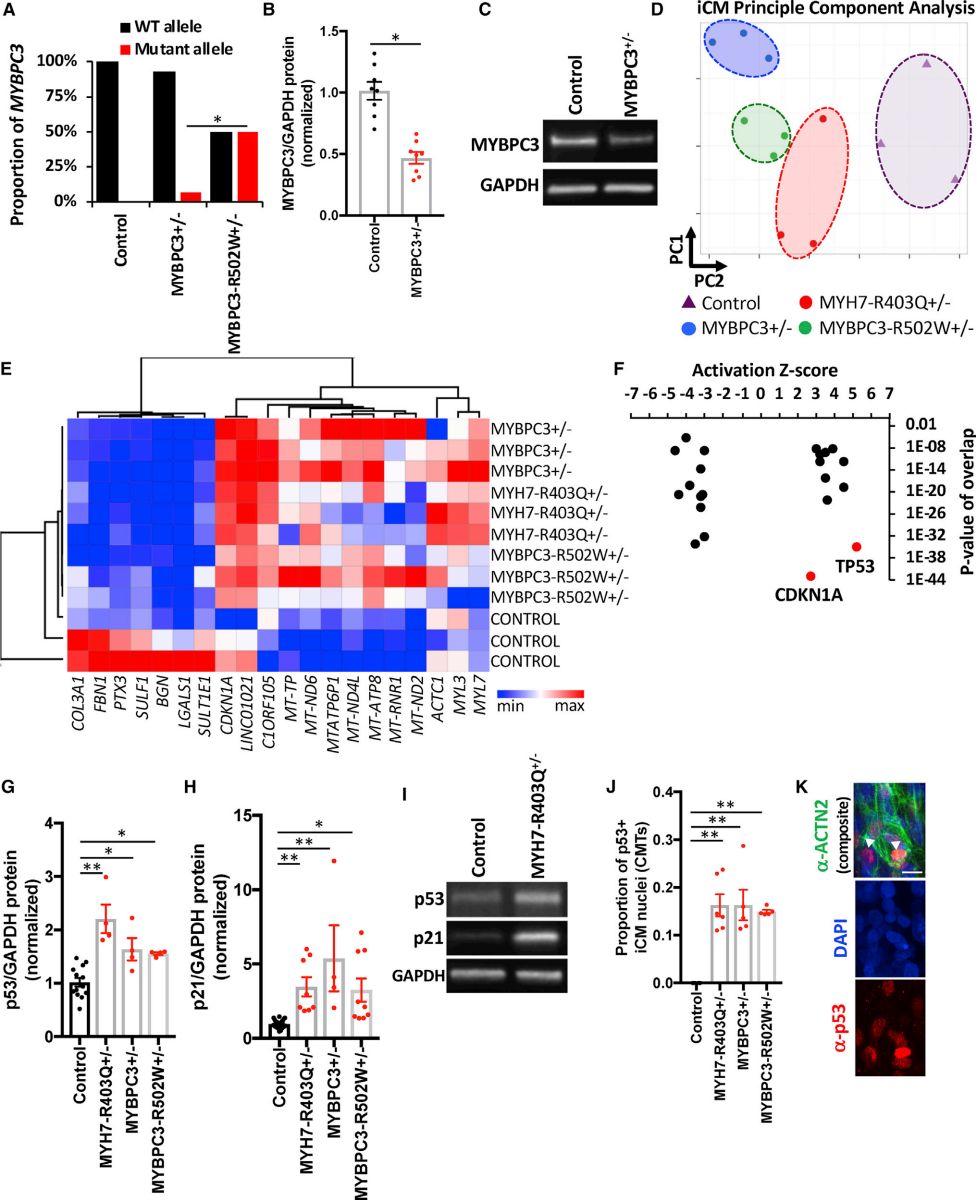
RNA Sequencing of HCM and Isogenic Control iCMs (A) By allele-specific analysis of gene transcripts obtained from isogenic control, MYBPC3^+/−^, and MYBPC3-R502W^+/–^ iCMs; MYBPC3 transcripts were quantified for control (wild-type [WT]; black bar) and mutant (red bar) alleles and shown as the proportion of total MYBPC3 expression. (B) Densitometry of immunoblots from protein lysates derived from control and MYBPC3^+/−^ iCMs, and probed for MYBPC3 (note: truncated MYBPC3 was not identified) and for protein loading with GAPDH. (C) Representative immunoblot from (B). (D) Principal-component analysis (PCA) of RNA transcripts from three biological replicates of isogenic control (purple triangles) and MYBPC3^+/−^ (blue circles), MYBPC3-R502W^+/–^ (green circles), and MYH7-R403Q^+/–^ iCMs (red circles). (E) Hierarchical clustering of genes contributing to PC1 and PC2 from (D) and illustrated by heatmap (Table S2). (F) Differentially expressed gene transcripts (log_2_FC > 0.3 or < –0.3 and false discovery rate < 0.1) were analyzed by pathway analysis using Ingenuity Pathway Analysis and identified pathways (black and red dots) were organized by activation *Z* score and p value of overlap. (G–I) Densitometry of immunoblots from control and HCM iCM protein lysates, normalized for protein loading (GAPDH) (G) and probed with antibodies to p53 or (H) p21. See representative blots in (I). (J and K) Quantification of iCM p53+ nuclei from confocal images of fixed CMTs immunostained with an antibody to p53 (red), cardiomyocyte-specific ACTN2, and DAPI co-stain (J). See representative image in (K), arrowhead marks p53+ nuclei that co-stain with ACTN2. Scale bar, 15 μm. Significance (^∗^p < 0.05 and ^∗∗^p < 0.001) was assessed by Fisher's exact test (A), Student's t test (B), or ANOVA (G, H, and J); and data are means ± SEM (error bars) (B, G, H, and J). Each data point represents a sample generated from a batch of iCMs (B, D, E, G, and H) or single CMT (J) generated by at least three biological replicates by iPS differentiation batch.

To identify molecular mechanisms of HCM mutations, we applied RNA sequencing to iCM samples from HCM mutations (MYH7-R403Q^+/–^, MYBPC3^+/−^, and MYBPC3-R502W^+/–^) and compared the results with isogenic controls. We started by analyzing the MYBPC3^+/−^ mutation, tryptophan-792-valine-fs, to determine how *MYBPC3* truncation mutations cause HCM. Consistent with nonsense-mediated messenger RNA decay, transcripts from the tryptophan-792-valine-fs allele were nearly absent compared with wild-type transcripts (Figure 5A). This is in contrast to the missense mutation R502W, which had no evidence of degradation of mutant transcripts. In parallel to changes in *MYBPC3* transcript level, cMyBP-C protein content was also reduced in MYBPC3^+/−^ (Figures 5B and 5C) but not R502W^+/–^ (Figures S4A and S4B) by immunoblotting iCM lysates with an antibody that recognizes the amino terminus of cMyBP-C proximal to the frameshift mutation. These data support a haploinsufficiency model of HCM-associated MYBPC3 frameshift mutations, and also that R502W is likely a loss-of-function missense mutation.

Next, we analyzed iCM gene transcripts by unsupervised principal-component analysis (PCA) to assess sample-to-sample distances. All HCM samples clustered distinctly from isogenic controls, while biological replicates within the same genotype clustered closely (Figures 5D and 5E; Table S3). Of note, gene components obtained from PC1 and PC2 include mitochondrial-encoded transcripts (*MT-TP*, *MT-ND6*, *MTATP6P1*, *MT-ND4L*, *MT-ATP8*, *MT-RNR1*, and *MT-ND2*), extracellular matrix-related transcripts (*COL3A1*, *FBN1*, *SULF1*, *BGN*, and *LGALS1*), sarcomere transcripts (*ACTC1*, *MYL3*, and *MYL7*), and p53-related gene targets (*CDKN1A* and *LINC01021*) (Hunten et al., 2015).

To better define the HCM-associated gene transcript program, we analyzed differentially expressed transcripts that were common to all HCM iCM models (Figure S4C; Table S4). In accord with the close proximity of HCM samples by PCA analysis, differentially expressed transcripts were also highly shared among HCM models. Of 1,050 total upregulated transcripts, 320 were shared by at least two HCM models, and of the 1,177 total downregulated transcripts, 419 were shared by at least two HCM models. Of note, the HCM phenotype did not correlate with expression of cardiac chamber-specific markers such as myosin light chain 7 (*MYL7*), myosin light chain 2 (*MYL2*), and potassium channel *HCN4* (Figure S4D). We then used differential expression results to perform pathway analysis in HCM iCM models using Ingenuity Pathway Analysis. Both p53 and CDKN1A (p21) pathways were predicted to be highly activated in HCM iCMs (Figure 5F; Table S5) as prioritized by activation *Z* score and p value of overlap. In parallel to transcript levels, p53 and p21 protein levels were increased in HCM iCM lysates (Figures 5G–5I). In addition, fixed and immunostained HCM CMTs also exhibited increased p53 staining within the nuclei of ACTN2+ iCMs (Figures 5J and 5K). Unexpectedly, these results implicate altered p53 signaling as a common molecular consequence of thick-filament HCM mutations.

### HCM iCMs Exhibit a p53-Dependent Cytotoxicity Induced by Metabolic Stress

**Figure 6:**
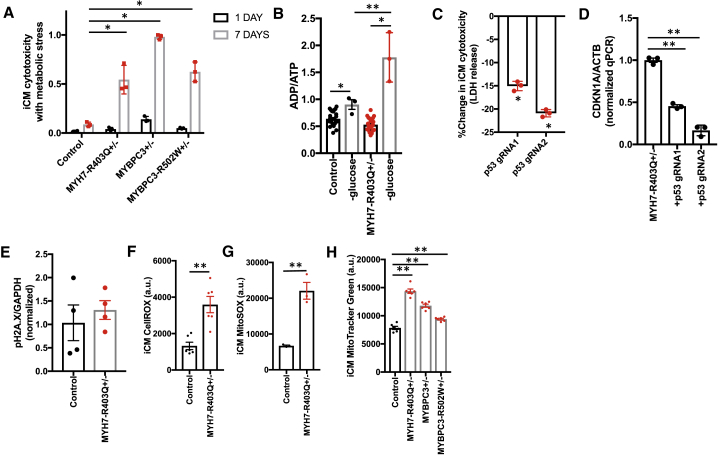
HCM iCMs Exhibit a p53-Dependent Cytotoxicity with Metabolic Stress that Is Related to Increased Oxidative Stress (A) Proportion of iCM death after 1 and 7 days of metabolic stress induced by growth in glucose-free medium. (B) ADP:ATP for iCMs cultured in normal growth medium and after 24 hr in glucose-free medium. (C) Change in iCM cytotoxicity measured by lactate dehydrogenase (LDH) release assay upon p53 genetic knockdown using lentiCRISPR encoding two independent gRNAs that target the *TP53* gene. (D) qPCR analysis of *CDKN1A* normalized to *ACTB* obtained from cDNA libraries generated from MYH7-R403Q^+/–^ iCMs transduced with lentiCRISPR encoding two independent *TP53* or non-targeted gRNAs. (E) Quantification of phosphorylated H2A.X normalized to GAPDH using densitometry analysis of immunoblots from iCM lysates. (F and G) Quantification of fluorescence (arbitrary units) using FACS analysis of iCMs stained with CellROX green (F) or MitoSOX red (G), a mitochondrial-specific probe for reactive oxygen species. (H) Quantification of fluorescence (arbitrary units) using FACS analysis of iCMs stained with MitoTracker green. Significance (^∗^p < 0.05 and ^∗∗^p < 0.001) was assessed by ANOVA (A) or Student's t test (B–H); and data are means ± SEM (error bars) (A–H). Each data point represents results obtained from a sample generated from a batch of iCMs (A–H) generated by at least three biological replicates by iPS differentiation batch.

To understand the role of elevated p53 signaling in HCM iCM models, we first determined whether p53 activation reflected increased iCM stress. We started by re-examining RNA sequencing data for p53-dependent gene expression changes that function in the regulation of cell death including *BBC3*, *BAX*, and *FAS*. HCM iCMs have increased expression of these transcripts relative to controls (Han et al., 2001). We then tested whether HCM mutations resulted in increased iCM death in normal growth conditions (Figure S4E). We could identify no baseline change in iCM death, which was also consistent with our observation that CMT cell number and tissue cross-sectional area were not altered by HCM mutations. We then considered whether HCM iCMs may be more susceptible to cell stress. Informed by the clinical observation that HCM patients have bio-energetic deficits *in vivo* (Crilley et al., 2003), as well as the increased expression of mitochondrial gene transcripts in HCM iCMs (Figure 5E), we measured iCM cell death induced by metabolic stress from glucose removal that has been used by others to induce energy stress (Jones et al., 2005). We found that HCM iCMs exhibited elevated cytotoxicity (Figure 6A) compared with controls, which was associated with increased ADP:ATP (Figure 6B)—a marker of metabolic stress. We next tested whether p53 knockdown by lentiCRISPR could rescue HCM-associated cytotoxicity induced by metabolic stress. P53 knockdown partially rescued HCM cytotoxicity induced by metabolic stress (Figure 6C), and also resulted in reduced expression of p53-target CDKN1A (Figure 6D).

To gain insights into molecular linkages between thick-filament HCM mutations and p53 activation, we started by measuring known regulators of p53, including DNA damage (Lakin and Jackson, 1999) and oxidative stress (Hwang et al., 2001). While the levels of phosphorylated histone 2A member X (Figure 6E), a marker of DNA damage was not different between R403Q^+/–^ iCMs compared with controls, reactive oxygen species (ROS) levels were increased over 3-fold (Figure 6F). Because ROS generation is generated by respiring mitochondria, we also stained iCMs with MitoSOX (Figure 6G) and MitoTracker dyes (Figure 6H) to quantify both mitochondrial-derived ROS and mitochondrial content, respectively. Compared with controls, HCM iCMs had higher mitochondrial-derived ROS production and mitochondrial content. Finally, we tested whether ROS inhibition by n-acetylcysteine (NAC) or p53 inhibition by pifithrin-α could normalize CMT force production in HCM CMTs. While pifithrin-α treatment had no influence on CMT twitch force, NAC treatment increased twitch force similarly in HCM and control CMTs. These data support that thick-filament HCM mutations result in hypercontractility independent from p53 activation and ROS production, and ROS is a negative regulator of CMT twitch force. In summary, we conclude that HCM mutations result in a state of contraction stress characterized by oxidative stress, p53 activation, and increased p53-dependent cytotoxicity in the setting of metabolic stress.

## Discussion

The genetic basis of HCM, most commonly due to thick-filament sarcomere mutations, was identified decades ago, yet the mechanisms that link sarcomere gene mutations with the HCM phenotype still remain unclear, in part because of the lack of a robust human *in vitro* model system to interrogate HCM pathogenesis. Here, we engineered four human HCM models using CRISPR to generate isogenic mutations in two of the most commonly mutated sarcomere genes, and measured the mechanical and molecular consequences of these variants in a biomimetic CMT assay. We sought to determine the role of genetic heterogeneity on phenotypes identified in CMT and iCM functional assays. Our study demonstrated a convergent model of HCM pathogenesis whereby four thick-filament HCM mutations, irrespective of mutation localization, induced a state of hypercontractility due to both increased twitch and resting tensions in parallel with delayed relaxation kinetics. The increased resting tension has particular importance as this component of sarcomere contraction is not affected by therapeutics that target only voltage-dependent factors, such as the L-type calcium channel, which are in clinical use for HCM patients, and provides a rationale for the identification of therapeutics such as direct myosin inhibitors that can reduce both resting and twitch contraction forces.

Our results of hypercontractility are in accord with recent functional studies of HCM-associated thin-filament mutant mouse models that support a sarcomere tension model (Davis et al., 2016). HCM mutations in thin-filament genes were found to increase tension primarily by increasing myofilament calcium sensitivity, as would be expected by changes in troponin complex function, while our findings are consistent with a model whereby thick-filament HCM mutations result in hypercontractility due to intrinsic changes in sarcomere function independent of changes in calcium delivery to the myofilament, and at least in part independent from changes in myofilament calcium sensitivity. Because thick-filament HCM mutations consistently increased iCM size, our study cannot exclude the contribution of iCM hypertrophy to hypercontractility, especially in relation to myofilament calcium sensitivity studies. Nonetheless, our model is also supported by recent biophysical studies of MHC-β and cMyBP-C interactions that report changes in the accessibility of myosin heads to generate force in the setting of HCM mutations (Nag et al., 2017). Our inability to observe previously described alterations in calcium handling may be due to the nature of the variants studied, non-cell-intrinsic mechanisms, or may be limited by the maturity state of all iCM studies. We propose that the calcium handling defects identified in other HCM studies are likely indirect consequences of sarcomere mutations such as induced by heart failure. In the future, it will be important to test other thick-filament variants to identify potential functional heterogeneity, as well as for comparison with thin-filament CMT phenotypes.

Our study illuminates how HCM-associated hypercontractility is maladaptive because it results in oxidative stress, which is associated with reduced iCM viability in the setting of metabolic stress *in vitro*. Energy imbalance in HCM patients with significant LVH has been identified in other studies (Jung et al., 1998), but to our knowledge our study is the first to implicate an early, cell-intrinsic metabolic vulnerability that results in altered cell survival. While we did not observe an energy imbalance in HCM iCMs in unstressed conditions, this may be secondary to the fetal nature of iCM metabolism that favors glucose rather than fatty acids for energy production. Our study also provides a new *in vitro* assay for screening therapeutics that modify HCM-associated iCM viability defects induced by metabolic stress, such as through genetic and chemical screens, which may provide new therapeutic targets for HCM patients. Finally, we identify that HCM mutations result in increased p53 signaling. While, to our knowledge, p53 has been well studied in the context of cancer and other disorders, it has not been implicated in HCM pathogenesis and only recently implicated in regulating the cardiac transcriptome (Mak et al., 2017). Notably, p53 genetic ablation also protected against heart failure due to pressure overload, but promoted age-associated cardiac dysfunction (Mak et al., 2017). Our results suggest that p53 ablation would be beneficial in HCM, and therefore molecular linkages between sarcomere variants, oxidative stress, and p53 activation, will need to be addressed in future studies with potential therapeutic implications. Finally, our robust genome-engineered platform using isogenic human iPSCs combined with CMT assays demonstrates a robust approach to interrogate HCM pathogenesis, test cardiomyopathy therapeutics, as well as classify sarcomere gene variants of unknown significance.

## Experimental Procedures

### iPSC Culture, iCM Differentiation, and Enrichment

The parental human iPSC line used for all studies was PGP1, a normal control line obtained from the Coriell Institute (GM23338) that has been described previously (Hinson et al., 2015, Hinson et al., 2016). This study was approved by the Jackson Laboratory institutional review committee and institutional review board approval was obtained as part of the Personal Genome Project. All iPSC lines were maintained on Matrigel (Corning)-coated tissue culture plates in mTeSR1 medium (STEMCELL). iPSC lines were screened for copy-number variants using Illumina SNP arrays as described previously (Hinson et al., 2015). iPSCs were differentiated to iCMs by sequential targeting of the WNT pathway as described previously (Lian et al., 2013). iCMs were maintained in RPMI with B27 supplement (Thermo Fisher Scientific) unless otherwise noted. iCMs were enriched by metabolic selection by previously described methods (Tohyama et al., 2013). On day 12 of iCM differentiation, beating iCMs were enriched by adding 4 mM DL-lactate (Sigma) in glucose-free DMEM medium (Thermo Fisher Scientific) for 48 hr. Following selection, enriched iCMs were maintained in RPMI with B27 supplement. Only differentiation batches with >90% troponin T2+ cells by fluorescence-activated cell sorting (FACS) or estimated by morphology were considered to be high purity for further assays. For all assays, iCMs were studied on differentiation days 30–35. For all iCM and CMT assays, at least biological triplicates (differentiation batches) were studied.

### CRISPR/Cas9 iPSC and iCM Gene Editing

Genetic modifications were generated using scarless iPSC clonal selection by following protocols described previously (Hinson et al., 2015). For *MYH7* and *MYBPC3* mutation generation, PGP1 iPSCs were electroporated with pCAG-eGFP-2A-CAS9 plasmid (obtained from Addgene), a single-stranded mutation-specific 90mer oligonucleotide and an optimized guide RNA plasmid (Table S1). After 48 hr, GFP+ iPSCs were sorted by flow cytometry FACS, clonally expanded, and Sanger sequenced for genotyping. Guide RNAs were designed and optimized using *in silico* methods that reduce risk of off-target mutations (crispr.mit.edu). For *MYH7* R403Q gene editing, the corresponding region of *MYH6* was sequenced to verify no off-target *MYH6* mutation. For lentiCRISPR (v2) experiments (Addgene), protocols for guide RNA cloning and lentivirus production were obtained from published methods, and a multiplicity of infection of 3 was used (Sanjana et al., 2014).

### CMT Production and Force Measurements

CMTs were prepared as described previously (Hinson et al., 2015). Polydimethylsiloxane (PDMS) (Sylgard 184 from Corning) cantilever devices were molded from SU-8 masters, with embedded 1-μM fluorescent microbeads (carboxylate FluoSpheres; Thermo Fisher Scientific). PDMS tissue gauge substrates were treated with 0.2% pluronic F127 (Sigma) for 30 min to reduce cell-extracellular matrix interactions. iCMs were disassociated using trypsin digestion and mixed with stromal cells (human cardiac fibroblasts; single lot obtained from Lonza), which were pre-treated with 10 μg/mL mitomycin C (Sigma) to prevent cell proliferation. The number of stromal cells was 7% of the total cell population, which is the quantity necessary for tissue compaction. A suspension of 1.3 × 10^6^ cells within reconstitution mixture containing 2.25 mg/mL collagen I (BD Biosciences) and 0.5 mg/mL human fibrinogen (Sigma) was added to the substrate. We measured CMT function at day 7 to allow for tissue compaction and stability of force generation. For quantifying tissue forces, fluorescence images were taken at 25 Hz with an Andor Dragonfly microscope (Andor iXon 888 EMCCD camera with HC PL Fluotar 5× objective mounted on a DMI8 [Leica] microscope that was equipped with a fully enclosed live-cell environmental chamber [Okolabs]). All tissues were biphasic stimulated at 1 Hz with a C-Pace EP stimulator (IonOptix) and platinum wire electrodes that were separated by 2 cm to the sides of the tissues tested. The displacement of fluorescent microbeads was tracked using the ParticleTracker plug-in in ImageJ (NIH). Displacement values were analyzed in Excel (Microsoft) to compute twitch force (dynamic force), resting tension, and kinetics. Resting tension was measured by subtracting the resting cantilever position from the cantilever position prior to tissue generation. Cantilever spring constants were computed using the empirically determined elastic modulus of PDMS and the dimensions of the tissue gauge device as described previously (Boudou et al., 2012). For small-molecule treatment, CMTs were treated in Tyrode's solution for 10 min with verapamil (Tocris), blebbistatin (Tocris), pifithrin-α (Tocris), or NAC (Sigma) prior to force measurements.

## Author Contributions

R.C., K.T., A.L., F.L., A.M.P., R.R., E.M., Y.-S.C., and J.T.H. designed and performed iPSC and iCM research, and analyzed and interpreted data. R.C., K.T., and J.T.H. wrote the manuscript. R.C. designed and engineered cardiac tissue devices. A.L., F.L., A.M.P., R.R., K.T., and R.C. generated cells for this study. K.A. and B.D.H. performed atomic force microscopy analysis of PDMS devices.

## Accession Numbers

Analyzed data are provided in supplemental tables. Genome sequencing datasets are deposited at the GEO under accession number GEO: GSE113907.

## Supplemental Information

Document S1. Supplemental Experimental Procedures and Figures S1–S5

Table S1. CRISPR, PCR, and Other Oligo Sequences

Table S2. CRISPR Off-Target and iPSC CNV Results

Table S3. Principal Components from iCM RNA Sequencing

Table S4. Differential Expression from iCM RNA Sequencing

Table S5. Pathway Analysis from iCM RNA Sequencing

Document S2. Article plus Supplemental Information
