# Supplementary material for: A Contraction Stress Model of Hypertrophic Cardiomyopathy due to Sarcomere Mutations
Source: Stem Cell Reports. 2018 Dec 13;12(1):71–83. doi: 10.1016/j.stemcr.2018.11.015 (PMC6335568; doi:10.1016/j.stemcr.2018.11.015)
Supplement: Document S1. Supplemental Experimental Procedures and Figures S1–S5 [file mmc1.pdf]

Stem Cell Reports, Volume *12*

Supplemental Information

A Contraction Stress Model of Hypertrophic Cardiomyopathy due to Sarcomere Mutations

Rachel Cohn, Ketan Thakar, Andre Lowe, Feria A. Ladha, Anthony M. Pettinato, Robert Romano, Emily Meredith, Yu-Sheng Chen, Katherine Atamanuk, Bryan D. Huey, and J. Travis Hinson

Title: A contraction stress model of hypertrophic cardiomyopathy due to sarcomere mutations

Authors: Rachel Cohn M.S.[1#], Ketan Thakar Ph.D.[1#], Andre Lowe M.S.[1], Feria Ladha M.S.[2], Anthony M. Pettinato B.S.[2], Robert Romano B.S.[2], Emily Meredith B.A.[1,2], Yu-Sheng Chen Ph.D.[1], Katherine Atamanuk B.S.[3], Bryan D. Huey Ph.D.[4] and J. Travis Hinson M.D.[1,2]

Supplemental Experimental Procedures:

Calcium (Ca^{2+}) imaging using Fluo-4 AM and Indo-1 AM

CMTs were loaded with 4 μM Fluo-4 AM (ThermoFisher) for 60 minutes at 37°C in Tyrode's solution (140 mM NaCl, 5.4 mM KCL, 1mM MgCl2, 10mM glucose, 1.8 mM CaCl2 and 10mM HEPES pH 7.4 with NaOH). Following loading, tissues were washed three times in Tyrodes solution. Using a solid-state 50 mW 488 nm laser and GFP filter (emission 525± 50nm), fluorescence intensities (F/F_o) were obtained while pacing tissues at 1 Hz. All images were captured at 30 fps on an Andor Dragonfly multimodal microscope. For each tissue, at least four regions of interest (ROIs) were analyzed for changes in Fluo-4 intensity, with the resting fluorescence value F_o determined by the average of the first five frames of the video. Background intensity was subtracted from all values. For iCM resting and caffeine-induced calcium quantification, single iCMs were disassociated from monolayer differentiation batches using accutase (ThermoFisher). Disassociated iCMs were loaded with 5 mM Indo-1 AM (ThermoFisher) in Tyrodes solution. ICMs were analyzed using a FACSymphony A5 analyzer (BD Biosciences). After excitation with a 60 mW 355 nm laser, Indo-1 signal ratios were determined using a 450nm dichroic mirror and BUF395 and BUV496 filters. To determine baseline calcium concentration, iCMs were analyzed for 20 seconds and the average Indo-1 ratio was obtained. To determine caffeine-induced calcium levels, a stock of 20 mM caffeine (ThermoFisher) in Tyrodes buffer was subsequently added to reach a 10 mM caffeine concentration, and maximum Indo-1 ratio was determined instantaneously.

Immunofluorescence, protein quantification and sarcomere analysis

Protein lysates obtained from iCMs were solubilized in RIPA buffer followed by western blotting. For analysis of MYBPC3 protein levels, an antibody that recognizes an n-terminal epitope of MYBPC3 protein was generously provided by Samantha Harris(Harris et al., 2002). All other antibodies used for immunoblotting include p53 (DO-7; ThermoFisher), p21 (#2947; Cell Signaling), phospho- and total ERK (#4370 and #4695; Cell Signaling), phospho- and total AKT (#2965 and #4691; Cell Signaling), phospho- and total p38 (#4511 and #8690; Cell Signaling), phospho- and total JNK (#4668 and #9252; Cell Signaling), phospho- and total CAMKII (#12716 and #4436; Cell Signaling), pH2A.X (#9718; Cell Signaling), GAPDH (#5174; Cell Signaling) and TNNI3 (#186820; ThermoFisher). For immunofluorescence, iCMs and CMTs were fixed with 4% paraformaldehyde (PFA) for 10 minutes, permeabilized and stained with antibodies to cardiac alpha actinin (#9465; Abcam) with DAPI co-stain. All immunofluorescence imaging was completed using a solid-state laser confocal microscope (Andor Dragonfly). CMT sarcomere analysis was done according to established methods(Bray et al., 2008). To determine z-disk anisotropy, CMTs were fixed with 4% PFA, permeabilized and immunostained with antibodies directed against Z-disk protein cardiac alpha actinin. Confocal images were obtained and analyzed using a modified ridge detection algorithm in ImageJ (NIH) to produce spatial maps of sarcomere Z-disks and the local orientation with respect to the x-axis of the image. For sarcomere length analysis, CMTs were relaxed in calcium-free buffer, fixed with 4% PFA, permeabilized and immunostained with antibodies directed against Z-disk protein

cardiac alpha actinin. Confocal images were analyzed in ImageJ (NIH) using a Z-disk profile plot of greater than three regions of interest per CMT. ICM cell area was measured in iCMs that were cultured on glass coverslips, fixed with 4% PFA, permeabilized, and similarly immunostained. ICM cell area were measured using ImageJ.

Cell viability, mitochondria and oxidative stress assays

For metabolic stress assays, iCMs were cultured at sub-confluence in 6-well plates coated with fibronectin (10μg/ml; Corning) at a density of 100,000 cells/well. Prior to metabolic stress conditions, cells were maintained in RPMI with B27 supplement. To induce nutrient stress, cells were maintained in glucose-free DMEM media (ThermoFisher) with B27 supplement for up to 7 days. To quantify iCM death, either LDH released from dead cells was analyzed from conditioned media using an LDH release assay (ThermoFisher), or viable cells were counted using live-cell imaging and a viability stain (MitoTracker Red CMXRos). ICM conditioned media was analyzed in a 96-well plate with a Synergy plate reader (BioTek) to quantify absorbance at 490 nm and 680 nm. For ADP/ATP ratios, 20,000 iCMs were plated on fibronectin-coated (10μg/ml; Corning) 96-well plates and analyzed by a bioluminescent ADP/ATP assay (Biovision). For mitochondrial content and reactive oxygen assays, iCMs were stained with MitoTracker Green (ThermoFisher) or MitoSOX Red (ThermoFisher) in accord with manufacturer's recommendations and single, disassociated iCMs were analyzed by FACS.

RNA sequencing, quantitative PCR and computational analyses

For iCM RNA sequencing experiments, total RNA was isolated from day 30-35 iCMs using Trizol (ThermoFisher). CDNA was constructed using Superscript III First-Strand synthesis (ThermoFisher). For each sample, total RNA from biological triplicates by differentiation batch was collected and sequenced. RNA sequencing libraries were generated using the TruSeq Stranded mRNA library preparation kit (Illumina). RNA sequencing libraries were sequenced on a HiSeq 2500 v4 SBS platform 2x100bp reads (Illumina). Sequences were aligned with STAR(Engstrom et al., 2013) to the hg38 human genome. For differential expression, DESeq2 (Bioconductor) was used. Heatmap analysis and hierarchical clustering were performed using the Morpheus tool (Broad Institute). For quantitative PCR analysis, iCM total RNA was isolated and processed as above. Gene-specific PCR primers were identified from the literature or designed using Primer3 (see table S1 for primer sequences) and transcripts were quantified using Fast SYBR Green (Applied Biosystems) on a ViiA7 Real-Time PCR system (Applied Biosystems). RNA sequencing results files have been deposited in GEO (GSE113907).

AFM Modulus Quantification

To measure the elastic modulus of the PDMS used for tissue gauge production, indentation measurements were performed using Atomic Force Microscopy (AFM) (Asylum Research MFP-3D, Santa Barbara, Ca). Using a colloidal silica spherical probe (AppNano FORT SiO_2-A), a 16x16 array of force-distance curves was acquired evenly spaced across a 2x2μm area for each measurement. Before and after these force-volume experiments, identical force-distance measurements on an incompressible surface were performed with a steel disk to calibrate the system sensitivity and to determine the spring constant for the force-transducing cantilever (1.75 nN/nm of deflection, within the range of specifications for these commercial probes). All 256 force-distance curves for each location studied were analyzed based on the Johnson-Kendall-Roberts model of elastic contact. Such JKR mechanics accommodate large adhesion energies between an indenting probe and sample(Gupta et al., 2007; Huey, 2007), both expected and observed for these studies with PDMS. This incorporates the following experimental parameters: the indenter possesses a spherical tip geometry with a radius of 2.5 μm; the probe modulus

appropriate for the colloidal silica sphere is 68.0 GPa; and this probe exhibits a Poisson ratio of 0.19. A Poisson ratio of .33 is conventionally assumed for the specimen. For each of the samples, force-volume maps were completed at three representative locations. The average elastic modulus from the resulting 768 individual indentations per specimen is reported.

Supplemental Figures:

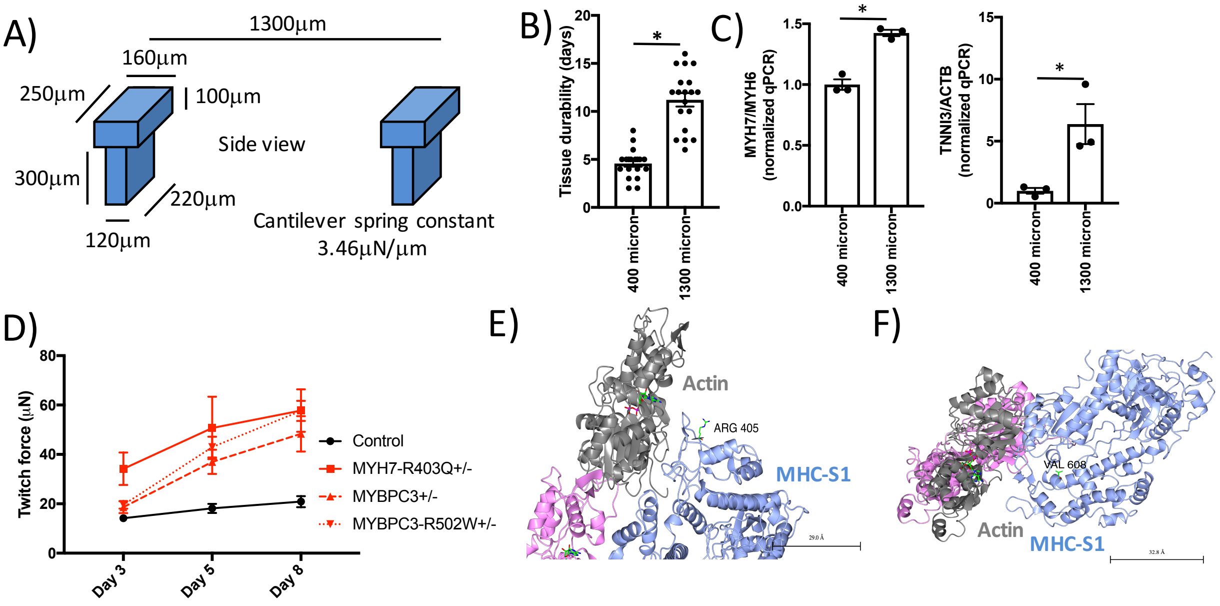

Figure S1. CMT assay to study HCM models. (A) Design of cantilevers is shown with xyz dimensions. **(B)** New cantilever design and larger CMTs resulted in improved tissue durability as measured by tissue survival. **(C)** Sarcomere gene expression (left panel-*MYH7* normalized to *MYH6* and right panel-*TNNI3* normalized to *ACTB* loading control) was measured using quantitative PCR between cDNA libraries produced from tissue samples of 400 μm and 1300 μm size. **(D)** Maximum twitch force plateaus for CMT models after day 5. **(E)** Ribbon diagram from electron cryomicroscopy structure of rabbit actomyosin rigor complex (myosin heavy chain S1 (MHC-S1) colored blue and actin monomers colored gray and pink) showing location or R403Q (equivalent to R405 in rabbit MHC)(Fujii and Namba, 2017). **(F)** Ribbon diagram from electron cryomicroscopy structure of rabbit actomyosin rigor complex showing location or V606M (equivalent to V608 in rabbit MHC)(Fujii and Namba, 2017). Significance was assessed by Student's t-test (B, C; *all $p<0.05$); data are means +/- SEM (error bars) (B, C and D). Scale bars are 29Å (E) and 32.8Å (F). Each data point represents a single CMT (B) or a sample generated from a batch of CMTs (C, D) produced by at least three biological replicates by iPS differentiation batch.

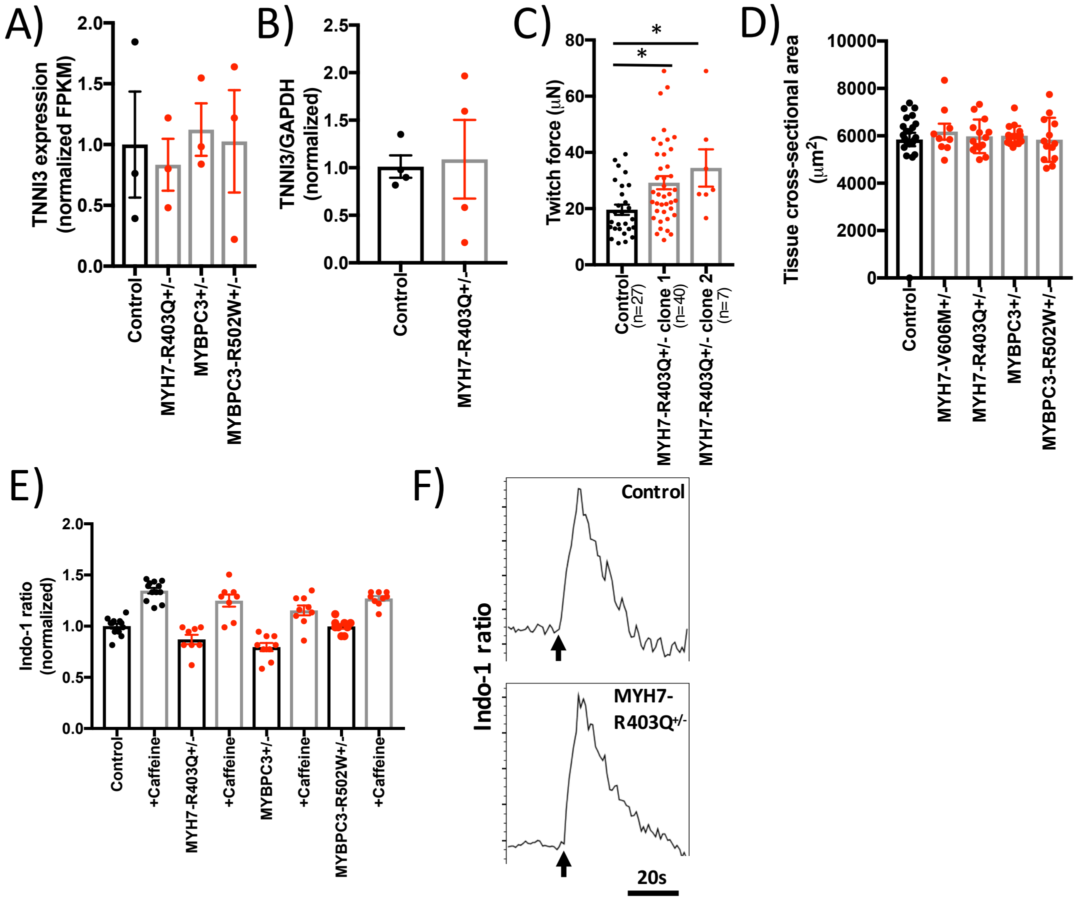

Figure S2. Characterization of HCM CMT models. (A) *TNNI3* normalized expression by RNA sequencing. **(B)** TNNI3 protein levels quantified by immunoblotting and densitometry analysis and normalized to GAPDH. **(C)** Maximum twitch force of CMTs generated from two independent clones of MYH7-R403Q$^{+/-}$ compared to isogenic controls. **(D)** Tissue cross-sectional area measured by confocal microscopy of four HCM models compared to isogenic controls. **(E)** Normalized basal and caffeine-induced calcium levels using FACS analysis to quantify Indo-1 ratiometric signal for HCM and control iCMs. **(F)** Representative FACS tracing of Indo-1 ratiometric signal for isogenic control or MYH7-R403Q$^{+/-}$ iCMs (black arrow denotes time of caffeine stimulation). Significance (*p<0.05 and **p<0.001) was assessed by FDR (A), Student's t-test (B) or ANOVA (A, C, D, and E); data are means +/- SEM (error bars) (A-E). Each data point represents a sample generated from a batch of CMTs (A, B) or a single CMT (C-E) produced by at least three biological replicates by iPS differentiation batch.

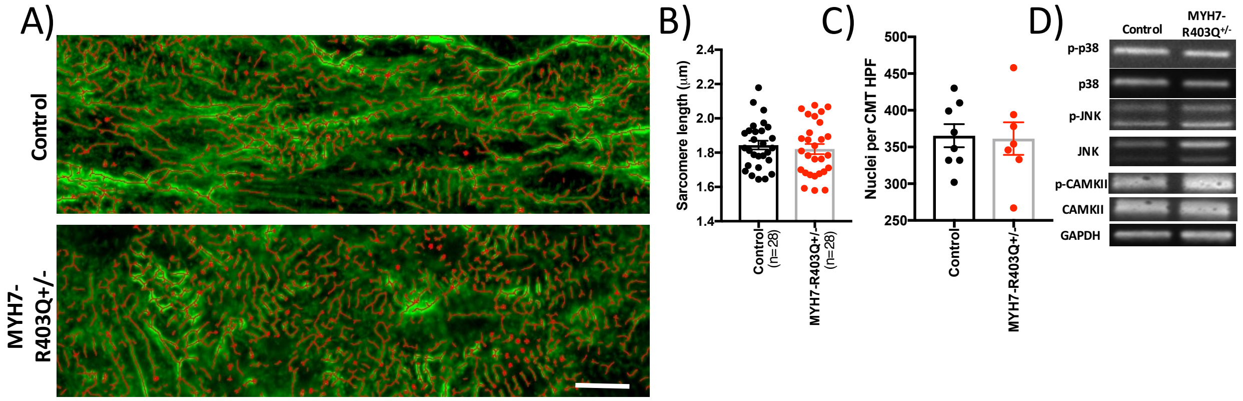

Figure S3. Sarcomere structure and functional analysis of HCM CMTs and iCMs. (A) Representative images of fixed CMTs immunostained with antibodies to cardiac alpha actinin to decorate sarcomere Z-disks (green) and analyzed by a modified ridge detection algorithm. Representative ridges shown in red with overlay confocal images (scale bar=10 μm). **(B)** Average sarcomere length obtained from confocal images of relaxed CMTs stained with antibodies to cardiac alpha actinin to decorate Z-disks. **(C)** CMT nuclei per high power field (HPF) of CMTs was quantified using confocal images of DAPI stained tissues. **(D)** Representative immunoblots from MYH7-R403Q$^{+/-}$ and control iCM lysates probed with antibodies to phospho- and total p38, phospho- and total JNK, phospho- and total CAMKII as well as GAPDH (loading control). Significance was assessed by Student's t-test (B and C); data are means +/- SEM (error bars) (B and C). Each data point represents a sample generated from a single CMT (B, C) produced by at least three biological replicates by iPS differentiation batch.

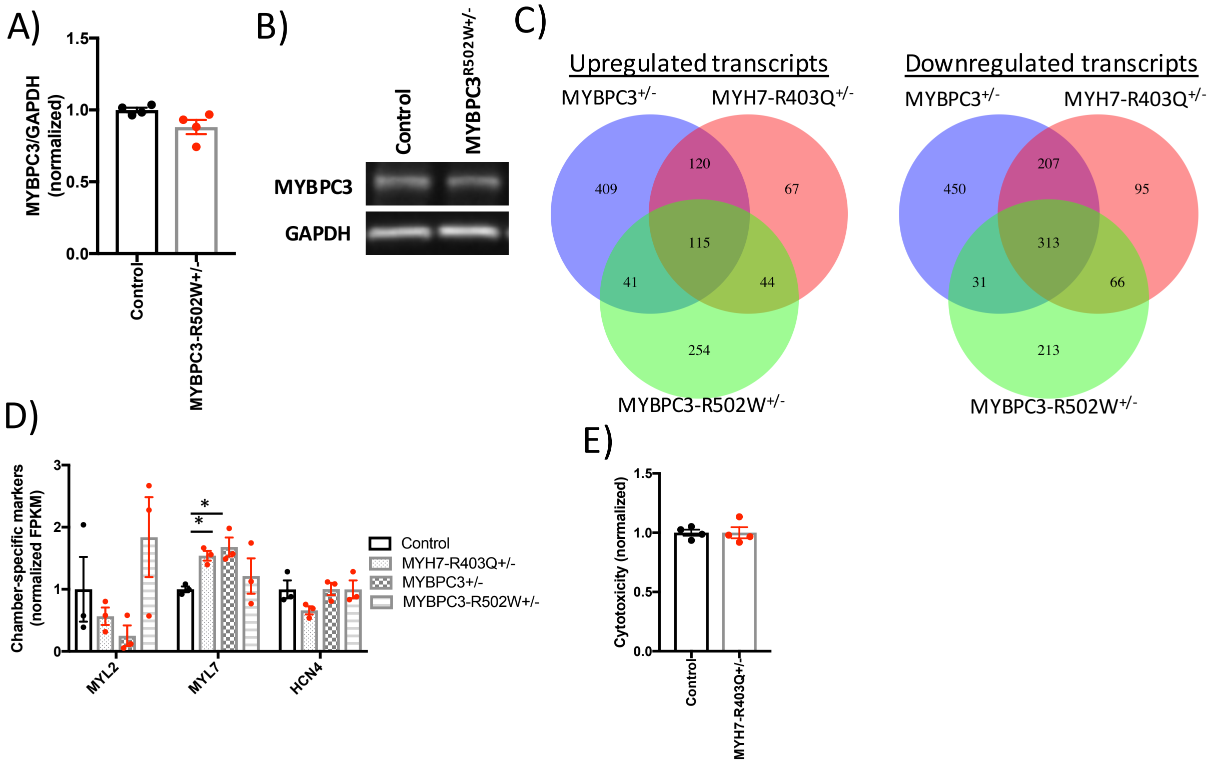

Figure S4. RNA sequencing of HCM models. (A) Quantification of MYBPC3 protein levels normalized to GAPDH by densitometry analysis of immunoblots of iCM lysates. **(B)** Representative MYBPC3 and GAPDH immunoblot used for (A). **(C)** Venn diagram to illustrate overlap in differential gene expression from RNA sequencing data obtained from three HCM models compared to isogenic controls (all FDR<0.10; $log_2FC>0.3$ or <-0.3; see table S4). **(D)** Transcript levels of cardiac chamber-specific genes as quantified by RNA sequencing. **(E)** Cytotoxicity of iCMs cultured in growth media. Significance (p<0.05) was assessed by Student's t-test (A, E) or FDR (C-D); data are means +/- SEM (error bars) (A, D and E). Each data point represents a sample generated from a batch of CMTs (A, C, D, E) produced by at least three biological replicates by iPS differentiation batch.

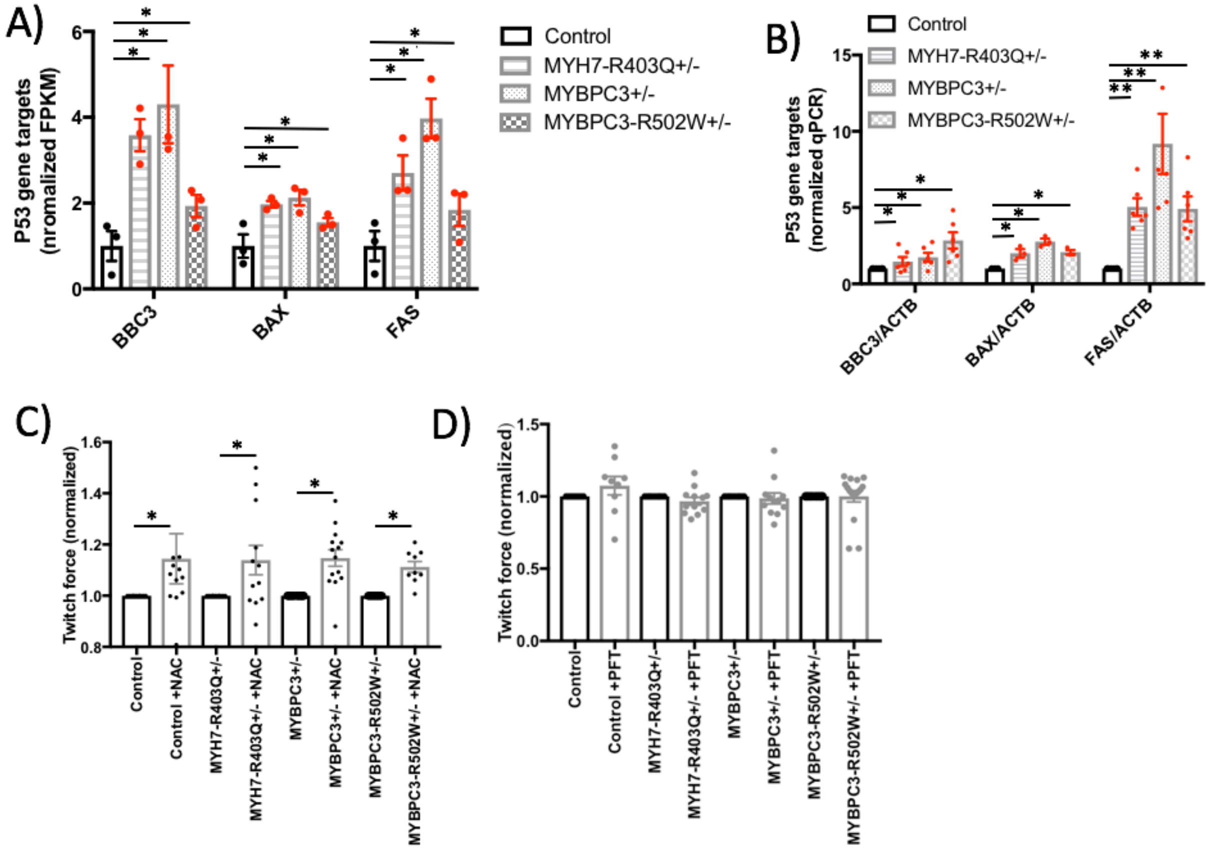

Figure S5. CMT force production after inhibition of p53 or oxidative stress using small molecules. (A) RNA-seq and **(B)** quantitative PCR analysis of p53 transcriptional targets in HCM compared to isogenic control iCMs. **(C)** Quantification of CMT twitch force after treatment with N-acetyl cysteine (NAC) compared to carrier control for HCM iCMs and isogenic controls. **(D)** Quantification of CMT twitch force after treatment with p53 inhibitor pifithrin-α (PFT) compared to carrier control for HCM iCMs and isogenic controls. Significance (*$p<0.05$) was assessed by FDR (A), ANOVA (B) and Student's t-test (C), and data are means +/- SEM (error bars) (A-C). Each data point represents a sample generated from a batch of CMTs (A, B) or a single CMT (C, D) produced by at least three biological replicates by iPS differentiation batch.

Captions for Tables S1-S5

Table S1. CRISPR, PCR and other oligo sequences. (A) Sequences for gRNA backbone, mutation-specific gRNA recognition site, ssODN HR donor sequence and Sanger sequencing primers for genome engineering experiments involving the *MYH7* and *MYBPC3* genes. **(B)** Summary statistics of CRISPR/Cas9 genome editing efficiencies in HCM iPS models including homology-directed repair (HDR) and non-homology end-joining (NHEJ) repair efficiencies. **(C)** LentiCRISPR experiments targeting *TP53* in iCMs including all gRNA sequences, oligonucleotides and qPCR primers for all gene expression studies.

Table S2. CRISPR off-target and iPSc CNV results. (A) List of *in silico* off-target CRISPR genomic loci that were predicted by crispr.mit.edu and sequencing primers to verify genotypes. **(B)** IPSc CNV analysis by virtual karyotyping using SNP arrays.

Table S3. Principle components from iCM RNA sequencing. Gene components and expression levels (fragments per million) of principle components 1 and 2 from fig.5D, E.

Table S4. Differential expression from iCM RNA sequencing. Upregulated and downregulated gene transcripts from DESeq2 analysis of biological triplicates from MYBPC3$^{+/-}$, MYBPC3-R502W$^{+/-}$ and MYH7-R403Q$^{+/-}$ compared to isogenic controls and organized by $\log_2$ fold change (>0.3 or <-0.3) and adjusted p-value (FDR cutoff <0.1).

Table S5. Pathway analysis from iCM RNA sequencing. Pathways enriched by Ingenuity Pathway Analysis of differential gene expression in HCM models compared to isogenic controls.

Captions for Movies S1-S2

Movie S1. Isogenic control CMT paced at 1Hz. Magnification- 5X. Scale bar=250μm.

Movie S2. HCM (MYBPC3-R502W$^{+/-}$) CMT paced at 1Hz. Magnification- 5X. Scale bar=250μm.

Supplemental references:

Bray, M.A., Sheehy, S.P., and Parker, K.K. (2008). Sarcomere alignment is regulated by myocyte shape. Cell Motil Cytoskeleton *65*, 641-651.

Engstrom, P.G., Steijger, T., Sipos, B., Grant, G.R., Kahles, A., Ratsch, G., Goldman, N., Hubbard, T.J., Harrow, J., Guigo, R., *et al.* (2013). Systematic evaluation of spliced alignment programs for RNA-seq data. Nat Methods *10*, 1185-1191.

Fujii, T., and Namba, K. (2017). Structure of actomyosin rigour complex at 5.2 A resolution and insights into the ATPase cycle mechanism. Nat Commun *8*, 13969.

Gupta, S., Carrillo, F., Li, C., Pruitt, L., and Puttlitz, C. (2007). Adhesive forces significantly affect elastic modulus determination of soft polymeric materials in nanoindentation. Materials Letters *61*, 448-451.

Harris, S.P., Bartley, C.R., Hacker, T.A., McDonald, K.S., Douglas, P.S., Greaser, M.L., Powers, P.A., and Moss, R.L. (2002). Hypertrophic cardiomyopathy in cardiac myosin binding protein-C knockout mice. Circ Res *90*, 594-601.

Huey, B.D. (2007). AFM and Acoustics: Fast, Quantitative Nanomechanical Mapping. Annual Review of Materials Research *37*, 351-385.
